# Quantitative Magnetization Transfer in In Vivo Healthy Human Skeletal Muscle at 3 T

**DOI:** 10.1002/mrm.22562

**Published:** 2010-07-27

**Authors:** Christopher D J Sinclair, Rebecca S Samson, David L Thomas, Nikolaus Weiskopf, Antoine Lutti, John S Thornton, Xavier Golay

**Affiliations:** 1 MRC Centre for Neuromuscular Diseases, UCL Institute of NeurologyQueen Square, London, United Kingdom; 2 Department of Brain Repair and Rehabilitation, UCL Institute of NeurologyQueen Square, London, United Kingdom; 3 National Hospital for Neurology NeurosurgeryQueen Square, London, United Kingdom; 4 Department of Neuroinflammation, UCL Institute of NeurologyQueen Square, London, United Kingdom; 5 Advanced MRI Group, Department of Medical Physics Bioengineering, University College LondonQueen Square, London, United Kingdom; 6 Wellcome Trust Centre for Neuroimaging, UCL Institute of NeurologyQueen Square, London, United Kingdom

**Keywords:** muscle, magnetization transfer, cross-relaxation, quantitative MRI

## Abstract

The value of quantitative MR methods as potential biomarkers in neuromuscular disease is being increasingly recognized. Previous studies of the magnetization transfer ratio have demonstrated sensitivity to muscle disease. The aim of this work was to investigate quantitative magnetization transfer imaging of skeletal muscle in healthy subjects at 3 T to evaluate its potential use in pathological muscle. The lower limb of 10 subjects was imaged using a 3D fast low-angle shot acquisition with variable magnetization transfer saturation pulse frequencies and amplitudes. The data were analyzed with an established quantitative two-pool model of magnetization transfer. *T*_1_ and *B*_1_ amplitude of excitation radiofrequency field maps were acquired and used as inputs to the quantitative magnetization transfer model, allowing properties of the free and restricted proton pools in muscle to be evaluated in seven different muscles in a region of interest analysis. The average restricted pool *T*_2_ relaxation time was found to be 5.9 ± 0.2μs in the soleus muscle and the restricted proton pool fraction was 8 ± 1%. Quantitative magnetization transfer imaging of muscle offers potential new biomarkers in muscle disease within a clinically feasible scan time. Magn Reson Med, 2010. © 2010 Wiley-Liss, Inc.

The value of MRI in the investigation of skeletal muscle affected by neuromuscular disease is increasingly being recognized, where objective and quantitative MRI measurements may be useful as biomarkers in trials of new therapies (1).

In MRI, the magnetization transfer (MT) describes the interactions of tissue–water protons residing in different macromolecular environments. In a simple two-pool picture, water protons in tissue are considered to reside in two independent environments, a “free” water proton pool that contributes to the conventionally visible MRI signal and a “restricted” proton pool in which the protons are bound to proteins and macromolecules. Exchange and cross-relaxation of magnetization between these two pools give rise to the MT effect, which has been widely studied in health and disease (2).

By selective saturation of the bound pool with applied off resonance radiofrequency (RF), MT can be used to generate additional contrast in conventional MRI images or may be used to make semiquantitative measurements of the MT ratio (MTR). The value of the MTR in studies of central nervous system (CNS) disease is well established largely due to the apparent association between underlying MT mechanisms and the extent of white matter myelination (3–5). A number of investigations in multiple sclerosis have prompted the adoption of MTR measures for monitoring treatment (6).

Muscle tissue also displays a prominent MT effect (7) and muscle MTR has been previously demonstrated to be reduced in the presence of myopathic processes. For example, the MTR of skeletal muscle of patients with limb-girdle muscle dystrophy was reduced in comparison with healthy control subjects (8), and MTR reductions have also been measured in skeletal muscle denervated in the neuropathic conditions Charcot-Marie-Tooth disease and chronic inflammatory demyelinating polyneuropathy, the latter showing association with clinically measured muscle strength (9). Furthermore, MTR reductions have been observed in ocular muscles involved in thyroid-related ophthalmopathy (10). MTR changes have also been noted in edema associated with dermatomyositis (11).

MTR measurements are inherently semiquantitative, influenced by a complex combination of MRI pulse sequence implementation and scanner hardware dependencies, such as the amplitude of static (polarizing) field, *B*_0_ and the amplitude of (excitation) radiofrequency field, *B*_1_, homogeneities as well as the intrinsic properties of the tissue under investigation such as the *T*_1_ and *T*_2_ relaxation times. These limitations prompted extensive efforts to establish models describing the MT process in more detail based on the presumed underlying physical principles. Such models can yield parameters such as the *T*_2_ relaxation time of the bound pool or the bound-pool fraction that are implementation independent. This field of “quantitative” MT (qMT) has yielded many promising avenues for clinical application, again, largely within the context of CNS disease, e.g., Refs. (4,12–14), dementia (15) and cancer (12) among others, despite its reliance on more complex acquisition protocols.

Despite some variations in the physical models and manner of incorporating experimental limitations, qMT provides a more rigorous generalization of the MTR approach and is well established in CNS applications. Therefore, the qMT technique is appropriate for exploitation in the context of muscle pathology with the potential to extract underlying pathology-related properties of muscle tissue in a manner less sensitive to the specific implementation, scanner hardware, and tissue relaxation properties than MTR measurements.

By applying qMT techniques to study muscle in a cohort of normal subjects, this work aims to bridge the gap between the promising sensitivity of MTR to muscle disease, already demonstrated, and the physical insight provided by qMT analysis. Before qMT methods can be applied to investigate muscle pathology, it is essential to develop clinically applicable optimized acquisition protocols and to understand the MT behavior of healthy muscle. Skeletal muscle in the lower leg of healthy individuals was investigated with a pulsed MT acquisition at 3 T in vivo, and the data were analyzed according to an established qMT model to extract the relevant physical parameters. Our purpose was to establish methods enabling the application of in vivo qMT measurements in neuromuscular conditions where measured qMT parameters might be useful as biomarkers.

## THEORY

### The Two-Pool Model

The most well-established physical description of the MT effect is the two-pool model first described by Henkelman et al. (16), in which the free proton pool (a) is coupled to the restricted proton pool (b) through magnetization exchange. Typically, the bound pool has a very short transverse relaxation time due to the binding of water protons to macromolecules and thus does not normally contribute to the NMR signal in conventional imaging. In the MT experiment, continuous wave (CW) or pulsed RF radiation is applied to selectively saturate the bound pool spins, permitting indirect interrogation of the bound pool spins due to the subsequent cross-relaxation toward equilibrium with the free pool protons.

The time dependence of the magnetizations of the free pool *M*^a^ and the bound pool *M*^b^ can be described by a set of Bloch equations that are modified to incorporate terms describing the magnetization exchange, the individual longitudinal and transverse relaxation rates of each pool, and the RF absorption rates of the free and bound pools, *R*_RF_a__ and *R*_RF_b__, respectively. In the steady state, all of the time derivatives of the magnetization vectors are assumed to be zero, allowing a set of coupled equations describing the longitudinal magnetization to be formulated (16). In this situation, the longitudinal component of magnetization of the free pool 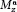, which has overall initial magnetization 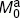, may be written as follows (16):


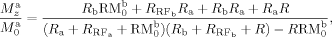
[1]

where *R* is the rate constant describing the magnetization exchange between the two pools. *R*_a_ and *R*_b_ in Eq. 1 are the longitudinal relaxation rates of the free and bound pools, respectively. The RF absorption rates *R*_RF_a,b__ are dependent on the absorption lineshapes *w*_a,b_(2πΔ), through the relationship *R*_RF_a,b__ = ω^2^π*w*_a,b_(2πΔ), where ω is the amplitude of the applied radiation (proportional to the applied *B*_1_ field) and Δ is the frequency of the applied radiation. The absorption lineshape of the free proton pool is a Lorentzian function given by (17):


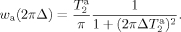
[2]

Ramani et al. (18) introduced the “restricted proton fraction *f* ” to the above formulation of the two-pool model, defined as


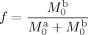
[3]

arguably providing a more intuitive physical interpretation of the relative pool sizes than the common alternative approach of normalizing 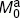 to 1 (16). By combining the expression in Eq. 3 with Eq. 1 and replacing *R*_RF_a__ with the expression given in Eq. 2, one obtains a formulation of the two-pool model, arranged by Ramani et al., describing the overall observed MR signal *S* in a given MT experiment (18):


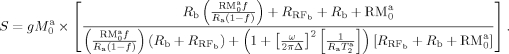
[4]

The parameter *g* is introduced above to describe a constant scanner and sequence-dependent scaling factor that determines the overall amplitude of the received signal. The signal described by Eq. 4 can be uniquely determined by considering the expression in terms of six combined parameters, namely 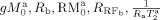, and 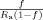 (18). The parameter 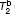 is incorporated via the absorption rate *R*_RF_b__, as described below. By experimentally varying ω and Δ to manipulate the MT response of the two pools, Eq. 4 may be fitted to the observed signal to obtain the above parameters.

The quantity *R*_a_ is explicitly required to obtain *f*. To obtain this, as noted in Ref.^16^, it is possible to make an independent measurement of *R*_aobs_, the experimentally measured longitudinal relaxation rate, from which *R*_a_ may be determined via the expression (16,18):


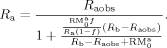
[5]

Therefore, an independent measurement of *T*_1obs_ = 1/*R*_aobs_ allows *f* to be uniquely determined.

### The Bound Pool Lineshape

It has been experimentally and theoretically shown that in the case of many biological tissues, a Lorentzian function does not adequately describe the RF absorption lineshape of the bound pool protons *w*_b_(2πΔ) (16,17,19). Of the alternative absorption profiles investigated, the Gaussian and super-Lorentzian functions have received the most attention. The latter has been shown to be most appropriate in studies of ex vivo animal muscle samples (19–21). The Gaussian function lineshape is described by 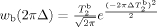 (19) and is considered most appropriate for dipole interactions in a rigid system, in contrast to the Lorentzian lineshape apparent in freely mobile liquid systems. In the intermediate regime of partially ordered materials, such as many biological tissues, the super-Lorentzian function, which includes an integration over all possible dipolar orientations in the semisolid material, is often most appropriate. The super-Lorentzian lineshape is given by:


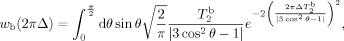
[6]

where θ is the dipole orientation angle with respect to the external magnetic field (17).

### Approximating Pulsed MT Saturation

The above descriptions of the steady state magnetizations of the bound and free pools hold only for CW irradiation. Several approaches have been used to compensate for the pulsed nature of MT experiments, necessary to limit RF power deposition in in vivo human studies, where exact analytical solutions of the modified Bloch equations are not available. These include the approach of Sled and Pike (20,22) in which the time evolution of the system is decomposed into exactly soluble components, the approach of Yarnykh (12) of neglecting the direct saturation of the free pool, and more recently the so-called minimal approximation of MT approach of solving the system numerically in small time intervals (23). In this work, we adopt the approach used by Ramani et al. (18) by treating the MT pulse as a rectangular CW signal with the same mean saturating power, *P*_sat_, as the experimentally used shaped pulse in each repetition time (24), the so-called CW-power equivalent (CWPE) approximation. The theoretical performance of particular MT model approximations have been compared systematically (23,25). The CWPE method has been applied a number of times in exploratory and clinical investigations (4,13,15,26,27) and has been established as reasonably robust in the brain (23,25). The CWPE equivalent frequency is given by (18,28):


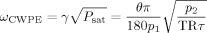
[7]

where τ is the pulse duration, θ is the nominal on-resonance effective flip angle in degrees, γ is the gyromagnetic ratio, and *p*_1_ and *p*_2_ are geometric factors describing the ratio of the area of the pulse to a rectangular pulse of the same amplitude and duration and the ratio of the mean square height of the pulse relative to a rectangular pulse of the same amplitude and duration, respectively (13,18,28).

## METHODS

Imaging was performed at 3 T (Siemens Magnetom TIM Trio, Erlangen, Germany) operated with the body transmit coil. Ten healthy subjects (five males, five females) between the ages of 26 and 55 with a mean age of 33.6 ± 8.7 years were recruited with the approval of the local ethical review committee. Subjects were positioned in the scanner feet-first and supine. Four elements of a surface matrix coil placed over the anterior and lateral surfaces of the lower limbs in combination with two elements of a spine matrix coil in the scanner bed received the signal from the right calf of all subjects. The center of all image volumes was prescribed to correspond with the point of widest circumference of the calf, encompassing the region of maximal muscle volume.

MT-prepared images were acquired using a locally implemented slab-selective spoiled 3D fast low-angle shot (FLASH) sequence [pulse repetition time/echo time (TR/TE) = 50/3 ms,α= 6°] allowing for a free choice of MT-saturation parameters including the pulse offset frequency, amplitude, shape, and duration (29). A flip angle of α = 6° was selected to receive sufficient signal while simultaneously minimizing *T*_1_ weighting. Images were acquired on a 128 × 128 × 16 matrix with a field of view (FOV) of 180 × 180 × 160 mm, frequency encoding in the L-R direction and with a parallel imaging acceleration factor of 2. To provide MT weighting, each RF excitation pulse was preceded by a 12 ms duration Gaussian shaped pulse of variable amplitude and frequency. To acquire a complete set of MT-weighted images for qMT analysis, the acquisition was repeated 14 times with distinct offset frequencies Δ of 1,2,5,10,20,50, and 100 kHz repeated at nominal flip angles of 350° and 500°. One of the 10 subjects was scanned with a TR of 51 ms due to scanner-specific absorption rate constraints.

Maps of *T*_1_ to determine *R*_aobs_ were obtained using a multiple flip-angle approach with a FLASH readout using the so-called DESPOT1 method described in Ref.^30^. Three sequential volumes with nominal flip angles of α = 5°, 15°, and 25°, respectively, and *TR* = 25 ms were acquired with the other imaging parameters and coverage set to match the 3D-FLASH MT sequence. The three volumes were coregistered, and the parameters 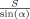 and 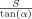 were calculated on a voxel-wise basis for the three flip angles α, where *S* is the gradient echo signal magnitude in each acquisition (30). The gradient *m* of a least-squares linear fit to 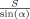 versus 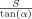 was used to calculate *T*_1_ in each voxel via the relationship 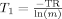 (30). The three nominal flip angles used in the calculation were adjusted to account for local *B*_1_ transmit deviations using *B*_1_ maps obtained as described below.

The spatial distribution of the *B*_1_ transmit field was evaluated using an optimized version of the actual flip angle imaging (AFI) method (31,32), using two nominal excitation pulses of 60° followed by delays TR_1_ and TR_2_ of 50 and 150 ms, respectively, and a gradient echo readout at TE = 3.05 ms. The actual flip angle α_AFI_ was calculated as 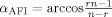, where 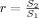, 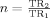, and *S*_1_ and *S*_2_ are the respective acquired signal magnitudes (31). The transverse field of view matched the MT sequence, sampled at the lower resolution matrix of 64 × 64, sufficient to sample the slowly varying spatial field. Nonselective excitation with 100% phase oversampling was used in the longitudinal direction to limit wrap-around artifacts to a region outwith the field of view of the MT acquisitions. The measured flip angle was normalized relative to the nominal flip angle of 60° and then maps of the field variation were obtained (31). These maps were subsequently used to correct the three nominal flip angles prescribed for the *T*_1_ mapping sequences prior to the calculation of *T*_1_ and to correct the nominal amplitudes of the applied MT pulses.

High-resolution *T*_1_-weighted images matching the central four partitions and the field of view of the MT weighted images were acquired for the purpose of delineating anatomical boundaries between different muscles, allowing the placement of regions of interest (2D turbo-spin echo, TR/TE = 600/9.9 ms, refocusing flip angle 130°, 512 × 512 matrix). These images also provided reference for coregistration of the MT, *T*_1_, and *B*_1_ data. Slice cross-talk and off-resonance MT effects due to the contiguous slices in the 2D-*T*_1_ w sequence did not affect the delineation of anatomical structures.

Total imaging time for the MT-weighted volumes, *T*_1_ and *B*_1_ maps was less than 15 min.

### Image Postprocessing and qMT Fitting Procedure

**FIG. 1:**
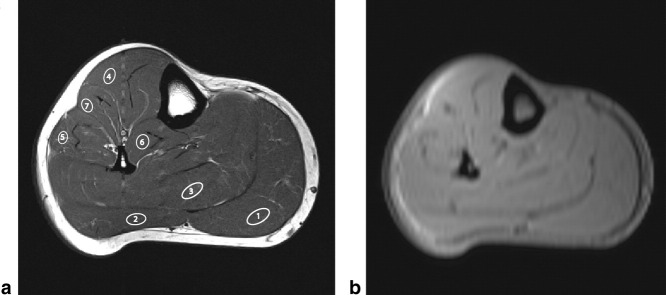
**a**: *T*_1_-weighted anatomical image of a single subject. Regions depicting the (1) medial head of the gastrocnemius, (2) lateral head of gastrocnemius, (3) soleus, (4) tibialis-anterior, (5) peroneus-longus, (6) tibialis-posterior, and (7) extensor digitorum muscles are drawn over the image. **b**: A typical MT-weighted image acquired with Δ = 20kHz and θ_nom_ = 350° using the 3D-FLASH sequence.

The data-set acquired from each of the subjects was analyzed as follows. Data were exported from the scanner onto a Dell Inspiron PC (3.16 GHz, 4 GB RAM) and processed using custom-written shell scripts. To account for any subject motion between the MT volume acquisitions and the *T*_1_ and *B*_1_ mapping, the four central slice partitions of each of the imaging volumes were registered to the anatomical *T*_1_-weighted volume using the linear registration FLIRT tool provided in the FSL software package (FSL, FMRIB, Oxford) (33). Maps of *T*_1_ and the *B*_1_ deviation were calculated on a voxel-wise basis using custom scripts written in MATLAB 7.6 (The Math Works, Natick, MA) (30,31). To determine the qMT parameters in various muscles, small region of interest masks were placed over the medial and lateral heads of the gastrocnemius muscles and in the central portions of the soleus, tibialis anterior, tibialis posterior, peroneus-longus, and extensor digitorum muscles on the *T*_1_-weighted images avoiding areas of fat and facia (Fig. 1a) using the FSLView software. The ROI masks were then transferred to the MT volumes, *T*_1_ and *B*_1_ maps and the mean and standard deviation of the MT-weighted signal, *T*_1_ and *B*_1_ deviations were calculated for each region. The mean *B*_1_ flip angle variation was used to correct the nominal MT pulse amplitudes, and the corresponding CWPE frequencies were calculated for each measured MT point according to Eq. 7 using *p*_1_ = 0.482 and *p*_2_ = 0.344 for the Gaussian MT pulse (18,28). The MT, *T*_1_, and *B*_1_ volumes were imported into Mathematica 7.0 (Wolfram Research, Champaign, IL) with the accompanying set of corrected flip angles and offset frequencies. The two-pool model of MT described in Eq. 4 was fitted to the set of 14 MT-weighted signal intensities with four free parameters and a maximum of 100 iterations using the nonlinear least squares fitting facility provided by the Mathematica 7 software which implements the Levenberg-Marquardt algorithm. In the fitting procedure, each point was weighted by the inverse of the variance of the MT-weighted pixel intensities in the ROI. The super-Lorentzian function, required for the fit, given in Eq. 6, was evaluated by numerical integration with 100 steps. The fitted model parameters and their standard errors were combined using Eqs. 3 and 5 to obtain the parameters 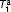, *f*, and 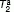 in each region. The standard deviation of the resulting parameters were obtained using standard procedures for compound propagation of uncertainties.

It has been widely noted that the parameter *R*_b_ is not well determined in fits to qMT models (16,18). It is a common practice to set this parameter to a constant value of 1s^−1^ to achieve a more stable solution without compromising the remaining fitted parameters (16,18,22,25). The same approach is adopted here.

The final two offset frequencies of the MT pulses used in the experiment (Δ = 50 and 100 kHz) were sufficiently far from resonance as to have little or no effect on either of the proton pools, resulting in no appreciable suppression of the MR signal due to magnetization transfer. Therefore, rather than explicitly fit for the parameter *g*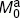, which is effectively the subject and scanner-specific measured MR signal amplitude in the absence of MT, the value of *g*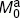 was determined directly from the data at the offset frequency of 100 kHz. With *g*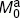 determined in this manner and with *R*_b_ fixed, there were thus four remaining free parameters to be fitted in the two-pool MT in Eq. 4.

The quality of the estimated models was evaluated by the root of the sum of the squared differences between the model and the data, normalized to *g*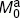 and divided by the number of degrees of freedom as well as the χ^2^ goodness-of-fit metric.

To establish representative values of qMT parameters in healthy individuals, as determined from the healthy subject cohort studied here, the mean value of each parameter for each muscle was calculated, weighted by the fit variance of each individual measurement. The weighted mean value and standard deviation across all subjects for each region were evaluated.

To obtain typical values for the MTR for each region examined, this was calculated as MTR (p.u.) = 100 × (*M*_0_ − *M*_1_)/*M*_0_ using the data obtained at θ_nom_ = 500° and Δ = 2 kHz for *M*_1_ and the fully relaxed points at Δ = 100 kHz for *M*_0_, corresponding parameters typically available in a clinical MTR acquisition. To obtain maps of qMT parameters for a given subject, exactly the same post-processing and fitting procedure described above was used on a voxel-by-voxel basis in Mathematica 7.0 (without any weighting of individual points) to produce spatial maps for each fitted parameter in a single subject.

## RESULTS

### Image Acquisitions

The 3D-FLASH MT sequence delivered good quality MT-weighted images with the signal-to-noise ratio in the unsaturated images consistently exceeding 300. An example of an MT-weighted image is given in Fig. 1b for one MT weighting in one subject (Δ = 20 kHz and θ_nom_ = 350°). For the Gaussian pulse and repetition time used in the acquisitions, the CWPE radial frequency was 304 and 434 rad/s for the 350 and 500° nominal flip-angle amplitudes, respectively.

**FIG. 2:**
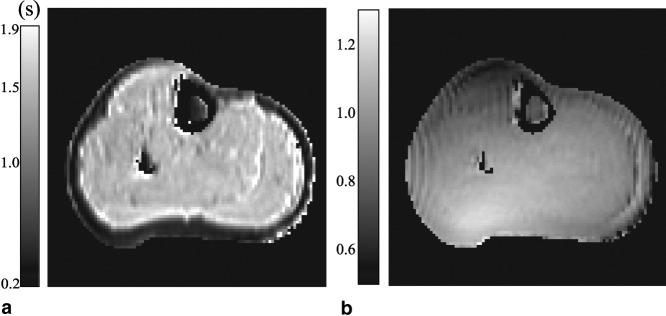
**a**: Map of *T*_1_ values in a single subject acquired using the multiple flip angle approach. **b**: Map of the *B*_1_ transmit field spatial variation in a single subject measured using the actual flip angle method. The values are expressed as fractional deviations from the prescribed nominal flip angle.

The anatomical *T*_1_-weighted image for the same subject is shown in Fig. 1a, with representative regions placed on the medial and lateral heads of the gastrocnemius muscle, soleus, tibialis anterior and posterior muscles, and peroneus-longus and extensor-digitorum muscles superposed. Examples of the *T*_1_ and *B*_1_ maps for the same subject are shown in Fig. 2.

### Performance of Model Fitting

**FIG. 3:**
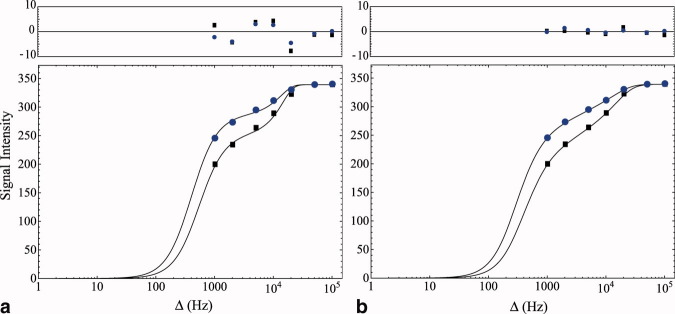
MT-weighted signal intensity plotted as a function of the MT pulse offset frequency for the two MT pulse amplitudes in the medial head of the gastrocnemius muscle of a single subject. Circles and squares: θ_nom_ = 350°,500°. Solid lines are a fit to the two-pool model signal equation. In (**a**), a Gaussian lineshape is used to describe the bound pool. In (**b**), a super-Lorentzian lineshape describes the absorption profile of the bound pool. The S-L lineshape provides a superior fit to the in vivo muscle data when compared with the Gaussian. The residual difference between the data and the fits are also plotted above the main graphs.

The MT-weighted signal amplitudes in a region of the medial head of the gastrocnemius muscle of a single subject are plotted on a log-linear scale in Fig. 3. Fits to four parameters of the two-pool MT model described by Eq. 4 are shown as solid lines. The residual difference between the data and fits are also shown. A Gaussian lineshape is used to describe the absorption profile of the bound pool in Fig. 3a, and the super-Lorenzian lineshape was used for the fit in Fig. 3b, as given by numerical integration of Eq. 6. The sum of squares deviation for the super-Lorentzian line shape in Fig. 3 is 4.5 times lower than the Gaussian lineshape, providing a superior fit to the in vivo muscle experimental data. This is in agreement with previous ex vivo studies of muscle (19–21). Because of the consistently superior fit quality of the super-Lorentzian line shape, it was used throughout the remainder of this work.

The same fitting procedure illustrated above was performed across subjects and across muscle regions.

Table 1 shows the fitted and derived qMT parameters in the soleus muscle for each of the 10 subjects examined. MTR and χ^2^ values for the individual fits are also shown.

**Table 1:** Values of the Four Fitted qMT Parameters and the Derived Quantities 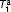, 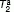, and *f* in the Soleus Muscle of the 10 Subjects Examined

	Age	Sex	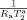	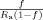	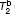 (μs)	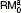 (s^−1^)	*T*_1obs_(s)	*T*_1*a*_(s)	*f*	*T* 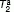 (ms)	MTR (p.u.)	χ^2^
1	26	F	48.3 ± 2.5	0.15 ± 0.004	6.01 ± 0.17	20.0 ± 3.5	1.49 ± 0.07	1.56 ± 0.08	0.085 ± 0.005	32.3 ± 1.7	36.7 ± 1.0	0.063
2	27	M	51.4 ± 2.9	0.14 ± 0.004	6.11 ± 0.18	19.6 ± 3.6	1.50 ± 0.09	1.57 ± 0.10	0.084 ± 0.005	30.5 ± 2.0	37.2 ± 1.2	0.020
3	29	M	55.4 ± 2.2	0.15 ± 0.004	6.02 ± 0.16	14.2 ± 1.9	1.44 ± 0.20	1.50 ± 0.20	0.088 ± 0.010	27.1 ± 3.1	34.1 ± 1.2	0.035
4	29	F	47.8 ± 2.3	0.14 ± 0.004	6.24 ± 0.19	14.3 ± 2.0	1.51 ± 0.06	1.58 ± 0.07	0.083 ± 0.004	33.0 ± 1.5	35.6 ± 1.5	0.048
5	29	M	53.5 ± 3.0	0.15 ± 0.004	5.76 ± 0.17	24.4 ± 5.7	1.50 ± 0.08	1.58 ± 0.09	0.089 ± 0.005	29.5 ± 1.6	35.2 ± 0.8	0.019
6	30	F	48.9 ± 2.4	0.14 ± 0.004	5.85 ± 0.18	21.4 ± 4.0	1.57 ± 0.09	1.65 ± 0.10	0.078 ± 0.005	33.7 ± 2.0	36.1 ± 1.6	0.029
7	33	F	46.1 ± 2.6	0.14 ± 0.004	5.91 ± 0.17	23.3 ± 4.2	1.56 ± 0.08	1.64 ± 0.09	0.078 ± 0.004	35.5 ± 1.9	38.6 ± 1.8	0.057
8	34	M	60.0 ± 3.9	0.16 ± 0.007	5.86 ± 0.22	14.8 ± 3.6	1.41 ± 0.08	1.47 ± 0.09	0.098 ± 0.006	24.6 ± 1.5	32.4 ± 1.1	0.070
9	40	F	48.8 ± 3.3	0.14 ± 0.005	5.80 ± 0.20	24.1 ± 6.7	1.55 ± 0.08	1.63 ± 0.09	0.081 ± 0.005	33.4 ± 1.8	34.3 ± 0.7	0.057
10	55	M	53.4 ± 3.0	0.16 ± 0.005	5.65 ± 0.20	20.8 ± 4.8	1.55 ± 0.20	1.63 ± 0.20	0.088 ± 0.009	30.6 ± 3.4	35.9 ± 1.2	0.045

Values of *T*_1obs_, MTR, and χ^2^ of the individual fits are also shown. M, male; F, female.

## DISCUSSION

Overall, the data acquired from the lower limb using the 3D-FLASH pulsed MT sequence withstood analysis using a quantitative model of magnetization transfer, providing good estimates of the model parameters for the healthy subjects studied in vivo. The two-pool model combined with the CWPE approximation of pulsed saturation and a super-Lorentzian RF absorption lineshape for the bound pool protons suitably described the behavior of the signal as a function of saturation frequency and amplitude with a good quality of fit.

Spatial inhomogeneities in the received signal due to distribution of surface array coil elements were compensated for inherently by the nature of the quantitative analysis. By mapping the transmit signal spatial variations, it was possible to measure the deviation from the nominally prescribed pulse flip angles and thus adjust the MT pulse saturation amplitudes appropriately.

The B_1_ maps acquired from each of the 10 subjects were qualitatively similar, demonstrating a slowly varying *B*_1_ spatial distribution. The greatest deviation below the nominal flip angle was in the anterior portion of the lower limb, localized around the border between the tibialis anterior muscle and the tibial bone. In this region, measured flip angles were about 40% lower than the nominally prescribed angle. Actual flip angles exceeded the nominal angle the most in the posterior portion of the limb, by approximately 15%. In addition to the adjustment of the pulsed MT saturation amplitudes, the *B*_1_ maps were also of importance for locally correcting the three flip angles used to map the *T*_1_ relaxation times. A 10% increase in the flip angle used in the qMT model causes a reduction of about 20% in the fitted bound pool fraction *f*. Equally, a 10% increase in the flip angles used to calculate *T*_1obs_ causes a 20% increase in *f*, indicating that both corrections are important in this model.

The *T*_1_ mapping approach, required to obtain *R*_a_ and *f*, yielded satisfactory results, after correction for local transmit field inhomogeneity. Measured *T*_1_ s were in reasonable agreement with previously reported measurements in skeletal muscle at 3 T (21,34) with mean values in the soleus muscle of 1.51 ±0.05 s, and the mapping method adequately fulfilled the required purpose of deriving *R*_a_ from the qMT fits in all cases.

### qMT Parameters

The four free parameters obtained directly from fitting the model to the qMT data were 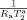, 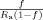 and 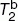 and R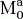. The parameter R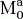 did not converge to discriminate values. It showed propensity to diverge to very large or small values. When convergent, the uncertainty on the fitted value was consistently large, indicating that this parameter is not sensitive to any process that might be useful in evaluating muscle. This behavior is in line with previous studies in the brain that have also found R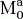 to be indiscriminate and widely varying in implementations of this model (4,18,26). However, as also previously noted, the other fitted parameters are not particularly sensitive to variations in R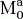 (26).

The parameter 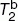, introduced through the bound-pool super-Lorentzian lineshape, showed very little variation between subjects indicating that the acquisition and fitting was not especially sensitive to this parameter. The mean value of 5.9 ± 0.2μs in the soleus muscle was somewhat smaller than in previous examinations of ex vivo samples of animal muscle that have been reported (summarized in Table 3). This may be due to differences in the hydration, perfusion, or local chemical environment in vivo or peculiar to human muscle. Equally, the particular formulation of the two-pool qMT model used here may be somewhat less sensitive to this parameter. Because of the important physical meaning of 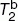, further investigations of the behavior of 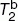 in other qMT model formulations (such as those in Refs. (12,22) and (23)) may be helpful.

**Table 3:** Summary of a Selection of Previously Reported Transverse Relaxation Times and Bound Pool Fractions in Muscle Samples

Ex vivo sample	*B*_0_	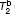 (μ*s*)	*f*	*F*	References
Bovine muscle	1.5 T	7.6 ± 0.3			(19)
Mouse skeletal muscle	1.5 T	8.2 ± 0.6		0.069 ± 0.016	(37)
Uncooked beef	1.5 T	6.6 ± 0.5	0.122 ± 0.009		(20)
Mouse skeletal muscle	3 T	8.7 ± 0.1		0.074 ± 0.013	(21)
Frog muscle	4.7 T			0.08 ± 0.01	(36)
In vivo human muscle	3 T	5.9 ± 0.2	0.08 ± 0.01		This work

Ex vivo experiments were performed with CW irradiation in contrast to this work. The parameter 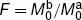 may be related to the parameter *f* used in this work via the expression *f* = *F*/(1 + *F*).

The model parameters 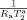 and 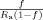 converged well in the fits, and the population mean values and standard errors reflected some of the expected variation between subjects. When combined with the measurements of *R*_aobs_ the parameters *f* and 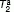 were obtained. Values of 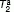 obtained from the healthy subjects using the qMT model were physically meaningful and closely reflected the established *T*_2_ values commonly observed in healthy muscle tissue (21,34) with a mean observed value of 31.0 ± 3.6 ms, providing substantial confidence that the qMT model used to describe muscle tissue here is a good physical description of the system.

The bound proton fraction *f* is perhaps arguably the most physically intuitive quantity to arise from the qMT modeling process and might potentially offer a useful MR measure with which to evaluate muscle pathology (3,5) that might be complementary to existing methods such as muscle biopsy. The mean value of *f* in the soleus muscle of the healthy subjects studied here of 0.084 ± 0.006 is in line with previously measured ex vivo animal tissue studies (20,21), indicating that around 8% of the MR-observable protons reside in the restricted pool bound to macromolecules or hydration layers. Whether this observed proton fraction is fundamentally determined at a cellular level or is better attributed to anatomical compartmentalization on a more macroscopic scale remains to be determined more precisely (8,36). Muscle fiber bundles are separated by connective tissue sheaths of perimysium and epimysium. Whether the bound proton fraction can be accounted for by such collagenous components, intra-or extra-myocellular lipids, or other tissue components merits further investigation. In CNS studies, where the bound proton fraction has been attributed to the degree of white matter myelination (5), this parameter has been singled out as a potential clinical marker of disease. The pathological specificity of such indices in muscle must be determined in studies of patient groups before similar conclusions on the use of qMT in muscle disease may be drawn. Nonetheless, the findings here in healthy subjects are a promising indication that qMT may be a valuable MR imaging tool in characterizing muscle.

There was not a great amount of variation in the measured parameters between subjects (Table 1). No clear trends relating to subject age were apparent though future studies may systematically examine the effects of subject demographics such as age on muscle qMT parameters. There was little anatomical variation in the MT parameters obtained across the different muscle regions examined (Table 2). A notable exception to this was the tibialis anterior muscle where fitted values deviated largely from the other regions. This region of the lower leg also corresponds to the area of greatest *B*_1_ variation (Fig. 2b). It is conceivable that although the *B*_1_ correction provides satisfactory compensation in most anatomical regions, enabling superior fit homogeneity, it performs less well in the anterior portion of the leg where the deviations are the greatest, thus providing systematic offset to the fitted model in this region. Physical parameters derived in this anterior portion of the leg must therefore be interpreted with caution.

**2:** Mean qMT Parameters for All Subjects Measured in Seven Muscle Regions (Expressed as Mean and Standard Deviation)

Region	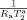	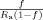	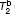 (μs)	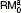 (s^−1^)	*T*_1obs_(s)	*T*1_a_(s)	*f*	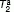 (ms)	MTR (p.u.)
Gastrocnemius-m	58.9 ± 6.0	0.16 ± 0.01	5.70 ± 0.18	13.9 ± 4.4	1.60 ± 0.08	1.70 ± 0.09	0.088 ± 0.005	30.7 ± 3.0	33.1 ± 2.1
Gastrocnemius-l	50.6 ± 6.1	0.14 ± 0.01	5.92 ± 0.20	15.7 ± 4.1	1.58 ± 0.08	1.66 ± 0.10	0.074 ± 0.007	33.0 ± 3.9	34.3 ± 2.3
Soleus	50.9 ± 3.9	0.15 ± 0.01	5.93 ± 0.17	17.0 ± 3.9	1.51 ± 0.05	1.58 ± 0.06	0.084 ± 0.006	31.0 ± 3.6	35.5 ± 1.9
Tibalis-anterior	67.3 ± 9.1	0.19 ± 0.02	5.99 ± 0.26	7.0 ± 2.3	1.52 ± 0.03	1.61 ± 0.05	0.099 ± 0.009	26.3 ± 2.3	26.7 ± 3.1
Peroneus-longus	54.7 ± 7.1	0.15 ± 0.02	6.07 ± 0.33	11.5 ± 3.4	1.39 ± 0.10	1.44 ± 0.10	0.087 ± 0.006	29.1 ± 2.4	31.4 ± 3.0
Tibialis-posterior	54.4 ± 5.9	0.15 ± 0.01	5.97 ± 0.20	13.2 ± 3.8	1.51 ± 0.05	1.59 ± 0.06	0.088 ± 0.005	30.2 ± 3.1	32.8 ± 2.5
Extensor-digitorum	63.8 ± 8.2	0.16 ± 0.02	6.15 ± 0.28	7.0 ± 3.1	1.47 ± 0.05	1.54 ± 0.07	0.091 ± 0.009	25.8 ± 2.5	27.4 ± 3.9

The MTR is calculated for Δ = 2kHz, θ_nom_ = 500° (ω_CWPE_ = 434rads^−1^). Key: gastrocnemius-m = medial head of gastrocnemius muscle; gastrocnemius-l = lateral head.

**FIG. 4:**
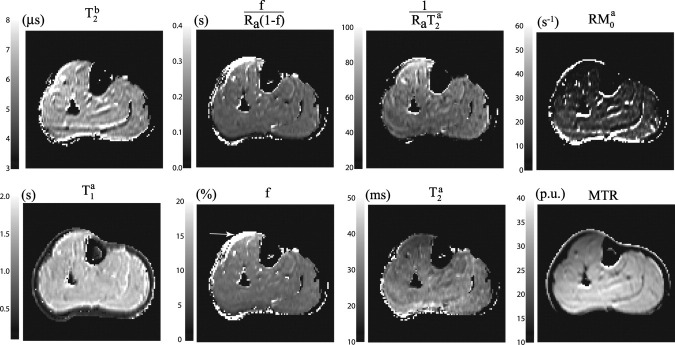
Parameter maps of the fitted and derived qMT parameters and MTR for a single subject. See text for discussion of arrow.

In the example parameter maps shown in Fig. 4, the maps generally show good homogeneity across the muscle tissue examined. Anatomical variations in such maps, if observed in patient groups, could prove useful as MR-derived measures of disease. The maps of 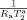, 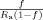, and the bound pool fraction, *f*, also show good homogeneity, with exception in the anterior portion of the leg, in the vicinity of the tibialis anterior muscle (see arrow Fig. 4). It seems likely that this deviation is again caused by inadequate *B*_1_ compensation in this region rather than a true anatomical variation. Nonetheless, with these caveats, qMT-derived maps of the muscle bound-pool fraction may indeed prove useful, especially in light of the previously observed sensitivity to disease of MTR, which is likely to be reflected in changes of qMT parameters.

The most detailed previous investigation of qMT parameters in muscle at 3 T focused on samples of excised murine skeletal muscle maintained at 37° and immersed in MR-compatible fluid (21). The investigators reported a 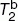 of 8.7 ± 0.1μs and a *T*_1_ value of 1.41 ± 0.01 s, measured independently by inversion recovery. In this work, 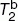 in the in vivo human soleus muscle was 5.9 ± 0.2μs and *T*_1_ was measured to be 1.51 ± 0.05 s using a variable flip angle method. These *T*_1_ measurements are in reasonable agreement, particularly considering the different acquisition methods and species. It is conceivable that *B*_1_ transmit variations may still contribute to the higher measured *T*_1_ values in this study, despite the steps taken to correct for this. The difference in 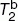 values is somewhat larger although some difference might reasonably be expected in view of the differing approaches to qMT acquisition used. The investigators in Ref.^21^ used a 7-s duration CW MT saturation pulse in combination with 182 independent signal amplitudes and off-resonant frequencies and four signal averages to obtain the qMT signal. In contrast, the constraints of acquisition time and RF deposition imposed by imaging human subjects in this work restricted acquisition to the use of 12-ms pulses and 14 independent qMT acquisition points. The consequent differences in the qMT modeling used and the underlying assumptions might reasonably lead to some difference in measured parameters. However, a previous systematic study of differences between pulsed and CW qMT modeling indicated that pulsed qMT modeling produces slightly larger rather than smaller estimates of 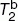 when compared with the CW case^23^. Given this observation, it seems likely that the in vivo human muscle measurements described here may yield a genuinely smaller 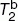 than in the previous investigations described in Ref.^21^ and in Table 3. However, given the additional factors of differing tissue environments, perfusion, and species used in each of these studies, the exact origin of observed differences in this work cannot be determined with absolute certainty. However, future investigations on ex vivo human muscle samples using CW irradiation is also likely to provide further useful insight into reconciling any remaining differences.

### Application

Imaging at 3 T field strength offers high signal-to-noise measurements when compared with lower field strengths; however a number of practical limitations at 3 T must be considered in volunteer and patient studies. Perhaps, most notable is the level of RF power deposition generated in MT-prepared sequences. The duty cycle of the sequence for a given MT saturation pulse amplitude and duration is restricted by the need to remain within regulatory scanner-specific absorption rate limits, thus limiting the dynamic range of applicable pulses and qMT acquisitions. This must be balanced against the requirement not to increase the repetition time sufficiently as to introduce significant *T*_1_ weighting in the MT acquisitions. Characteristic *B*_1_ inhomogeneities at 3 T must be carefully considered and accounted for with appropriate *B*_1_ evaluation techniques. No explicit *B*_0_ compensation was applied in this work because our own measurements of the *B*_0_ deviations in the lower leg (data not shown) reveal the deviations to be typically at least 100 times less than the smallest offset frequency used here (Δ= 1 kHz) and therefore make a negligible contribution to the model. However, *B*_1_ and *B*_0_ compensation may be combined in the future for further subtle optimization of the technique. With these considerations in mind, however, an accurate qMT evaluation of muscle is possible within the constraints imposed by clinical imaging.

A natural extension to this work would be to consider other well-established approaches to the qMT model and pulsed MT approximations (12,20,23), in particular their relative sensitivities to various muscle properties in healthy subjects or alternative sequence implementations (38). Further systematic mathematical optimization of the measurement scheme, such as the chosen offset frequencies and amplitudes, to specifically sensitize the acquisition to the tissue under investigation is possible based on prior knowledge of the MT parameters using the theory of Cramer-Rao lower bounds, and the parameters measured in this work could be used precisely for this purpose (39).

Based on the qMT results presented here and considering previous pertinent MTR results, the applicability of these methods to patient groups is very promising. Primary muscle conditions such as inflammatory myositis (40) involve a range of pathologies including edema-like changes and infiltration of fat and connective tissue over time, which may be expected to influence the observed magnetization transfer processes. Further investigation of muscular dystrophies with qMT techniques is also warranted. An additional important source of information in patient studies is the potential to correlate measured qMT parameters with muscle biopsy samples and histology findings, an approach already offering some further insight in CNS conditions (5).

## CONCLUSION

We have demonstrated that qMT imaging using an established two-pool model of MT can be successfully applied to human skeletal muscle in vivo. Normative values for the model parameters were determined in a group of healthy adults, providing a more complete characterization of the MT process in muscle than that afforded by measurement of the MTR alone. We have established an acquisition protocol that can be performed in a clinically acceptable timescale at 3 T. This work will greatly facilitate future studies that investigate the pathological dependencies of qMT parameters in patients with neuromuscular diseases. Encouraging early reports have demonstrated the sensitivity of MTR to myopathy. Our results suggest that the more rigorous qMT approach is both practical clinically and has the potential to provide objective quantitative markers of disease onset, progression, and response to therapy, which are urgently required in the context of neuromuscular medicine.
